# Can the Neutrophil-to-Lymphocyte Ratio, Platelet-to-Lymphocyte Ratio, and C-Reactive Protein-to-Albumin Ratio Always Predict Mortality in Acute Ischemic Stroke Patients Undergoing Mechanical Thrombectomy? A Single-Center Experience

**DOI:** 10.3390/brainsci15030323

**Published:** 2025-03-20

**Authors:** Şennur Delibaş Katı, Firdevs Ezgi Uçan Tokuç

**Affiliations:** Department of Neurology, University of Health Sciences, Antalya Training and Research Hospital, 07100 Antalya, Türkiye

**Keywords:** acute ischemic stroke, endovascular therapies, intracranial hemorrhage, mortality, neutrophil-to-lymphocyte ratio, platelet-to-lymphocyte ratio, hypersensitive C-reactive protein-to-albumin ratio

## Abstract

**Background**: Predicting mortality in patients with acute ischemic stroke who need endovascular treatment (EVT) has previously been shown to be related to inflammation. In this study, we aimed to examine the effects of the neutrophil-to-lymphocyte ratio (NLR), platelet-to-lymphocyte ratio (PLR), and hypersensitive C-reactive protein-to-albumin ratio (CAR) values on mortality and hemorrhagic transformation. **Methods**: A total of 225 adult patients who underwent EVT between 2022 and 2024 were retrospectively analyzed. The presence of intracranial hemorrhage (ICH) after the procedure; good and poor clinical outcomes according to modified Rankin Scores; mortality status; and NLR, PLR, and CAR values were collected. **Results**: The average age of the patients was 66.95 ± 12.74 years, and 133 (59.1%) patients were male. Thirty-eight (30.4%) patients had symptomatic ICH. While 164 (72.9%) patients had a poor outcome, 80 (35.6%) patients died. There was a correlation between the NLR and PLR values and symptomatic ICH (*p* = 0.013, 0.009, respectively) in the univariate analysis, but this relationship was not found in the multivariate analyses (*p*: 0.212 and *p*: 0.459). No statistically significant relationship was observed between the CAR and symptomatic ICH and mortality (*p* = 0.784, 0.079, respectively). When the laboratory data were compared according to the mortality status, the NLR and PLR were observed to be statistically significantly higher in the patients with mortality (*p* < 0.001, 0.005, respectively) in univariate analyses. But, as a result, the NLR, PLR, and CAR were not associated with ICH and mortality according to the multivariable logistic regression analysis. **Conclusions**: Our findings highlight the need to better understand the post-stroke immune response. Our study demonstrated that the NLR, PLR, and CAR were not associated with ICH and mortality according to the multivariable logistic regression analysis.

## 1. Introduction

Large vessel occlusion (LVO) is responsible for approximately 20% of acute ischemic stroke cases [1]. Endovascular therapies (EVTs) have been proven to be effective and are increasingly used in acute ischemic stroke caused by large vessel occlusion [2,3]. However, it is known that not every patient with successful reperfusion has a good clinical outcome. There may be many reasons for this. Conditions such as procedure time, technique, number of passes, and the presence of hemorrhagic transformation have a negative impact on mortality despite successful reperfusion [4]. Besides EVT-related causes, many studies have previously focused on the relationship between ischemic stroke and inflammation [5,6]. Studies have shown that the rapid activation of inflammation and immune response occurs as a result of the disruption of the blood–brain barrier after ischemic stroke [7]. As a result, it has been observed that there is a tendency for hemorrhagic transformation [8]. For this reason, laboratory parameters indicating inflammation have been searched for and the idea that different biomarkers can be used in this field has emerged. Some of these are the neutrophil-to-lymphocyte ratio (NLR), platelet-to-lymphocyte ratio (PLR), and hypersensitive C-reactive protein-to-albumin ratio (CAR), which are thought to predict mortality [9,10,11]. It is also important to determine these effects in an easy and cheap way.

Our stroke center accepts patients from many large districts and two surrounding provinces and consists of different patient groups. Most of the patients who come to our center and undergo EVT are followed up with in our Neurology Intensive Care Unit. In this study, we aimed to examine the effects of the NLR, PLR, and CAR values on mortality and hemorrhagic transformation in patients with acute ischemic stroke who underwent EVT.

## 2. Materials and Methods

### 2.1. Study Population and Study Design

All patients hospitalized in the Neurology Intensive Care Unit of Antalya Training and Research Hospital between February 2022 and August 2024 were retrospectively scanned. A total of 225 patients over 18 years of age who underwent EVT for internal carotid artery (ICA), middle cerebral artery (MCA), and basilar artery occlusion due to acute ischemic stroke and whose door-to-needle time was less than 24 h were included in the study. Door-to-needle time for anterior circulation stroke is a maximum of 6 h while it is 24 h for posterior circulation stroke. Medium vessel occlusions such as those of the posterior cerebral artery and anterior cerebral artery were not included in the study. We included patients who were hospitalized in the Neurology Intensive Care Unit of our hospital. The other patients who were referred to other intensive care units or hospitals were not included because of the lacking data during follow-up.

Patients with missing laboratory data (98 patients); patients for whom neuroimaging could not be performed after EVT (36 patients); patients with an infective condition before the procedure (24 patients); patients with a recent operation history (18 patients); patients with a history of malignancy (46 patients), hematological, or rheumatological diseases (16 patients); patients on immunosuppressive treatment (23 patients); and patients with a history of stroke within the last month (33 patients) were excluded from the study. Since a considerable number of patients did not have regular antiaggregant or anti-coagulant use, we could not use these data for statistical analysis.

The interventionalists are blinded to both the laboratory findings and clinical outcomes either.

### 2.2. Clinical Assessments

In our clinic, full blood, biochemistry and coagulation tests are routinely performed before EVT and within the first 24 h after EVT. Additionally, patients treated with EVT are followed up on with brain computerized tomography (CT) 4–24 h after the procedure. If clinical worsening is observed, brain CT is repeated. In addition, cranial magnetic resonance imaging is performed on all patients who do not have any objections to the imaging.

The following covariates were included in the analysis: demographic variables (age and gender), clinical parameters (NIHSS score, mRS score, and ASPECT score), stroke-related variables (LVO localization (ICA, MCA, and basilar artery), IV-tPA administration, and mTICI score), laboratory values (neutrophil-to-lymphocyte ratio [NLR], platelet-to-lymphocyte ratio [PLR], C-reactive protein-to-albumin ratio [CAR], hemoglobin, white blood cell count, platelet count, and albumin levels).

Medical data of 225 patients included demographic data, vascular risk factors, National Institutes of Health Stroke Scale (NIHSS), LVO localization, history of intravenous thrombolytic therapy (IV-tPA), Alberta Stroke Program Early CT (ASPECT) score, and modified Rankin Score (mRS), and data on modified Treatment In Cerebral Infarction (mTICI) scores were collected. mRS scores were divided into two groups: between 0 and 2 was categorized as good clinical outcomes and between 3 and 6 as poor clinical outcomes. The ASPECT scores of the patients were divided into 2 groups: 0–6 and 7–10. All these scorings were performed by one neurologist. Symptomatic intracranial hemorrhage (ICH) is defined as a worsening of ≥4 points in the NIHHS total score or ≥2 points in an NIHSS category, excluding other causes according to Heidelberg standards [12]. Hemoglobin, white blood cell count, neutrophil, platelet, lymphocyte, and monocyte numbers as well as CRP and albumin values were recorded from the blood samples taken from the patients. NLR was calculated as neutrophil/lymphocyte count, PLR as platelet/lymphocyte count, CAR as hypersensitive C-reactive protein/albumin value.

### 2.3. Statistical Analysis

Data were analyzed using IBM SPSS v23 and version 4.4.1 of the R programming language. Pearson’s Chi-square test and Yates’s correction were used to examine the relationship between categorical variables. In order to evaluate the effect of multiple independent variables on the dependent variable, univariate and multivariate analyses were conducted. The Mann–Whitney U test was used to compare non-normally distributed data according to binary groups, and the independent two-sample *t* test was used to compare normally distributed data. The cut-off value of the parameters to distinguish mortality and bleeding was examined by ROC analysis. The effects of independent variables on mortality and bleeding were examined by binary logistic regression analysis. Mean ± standard deviation and median (minimum–maximum) were used to display quantitative data. Frequency and percentage were used to display categorical data. The significance level was taken as *p* < 0.05.

## 3. Results

All patients hospitalized in the Neurology Intensive Care Unit of Antalya Training and Research Hospital between 2022 and 2024 were retrospectively analyzed. In total, 225 patients who underwent EVT due to acute ischemic stroke and who were eligible according to the appropriate criteria were included in the study.

The average age of the patients was 66.95 ± 12.74 years, and 133 (59.1%) patients were male. The median NIHHS was 15.15 (62.3%) patients received EVT alone, and 96 (37.6%) patients received IV-tPA together with EVT (Table 1). Type 1 hemorrhagic transformation was observed in 56 (24.9%) patients, type 2 hemorrhagic transformation was observed in 39 (17.3%) patients, parenchymal hematoma type 1 in 15 (6.7%) patients, and parenchymal hematoma type 2 in 15 (6.7%) patients. Symptomatic ICH was observed in 38 (30.4%) patients. A total of 80 (35.6%) patients died (Table 1).

When the patients were divided into two groups according to the presence of ICH, male gender was statistically higher in the ICH group (*p* = 0.013). It was also observed that the mRS scores were higher and death was observed at a higher rate in the ICH group (*p* = 0.007, <0.001, respectively) (Table 2). When the laboratory data of the patients with and without ICH were compared, the NLR values were observed to be statistically significantly higher in the ICH group (*p* = 0.015). No statistical difference was observed between the PLR and CAR values in the patients with and without ICH (*p* = 0.105, 0.815, respectively) (Table 3).

When the laboratory data were compared according to the mortality status, the NLR and PLR were observed to be statistically significantly higher in the patients with mortality (*p* < 0.001, 0.005, respectively) (Table 4). When the patients were grouped according to good and bad clinical outcomes according to the mRS, the NLR and PLR values were statistically significantly higher in the poor clinical outcome group (*p* < 0.001, *p* < 0.001, respectively). The CAR values did not differ for the good and poor clinical outcomes and mortality (*p* = 0.137) (Table 5).

The risk factors affecting the development of ICH were examined through a binary logistic regression analysis, and the effect of high NLR values on ICH was observed in univariate analyses (*p* = 0.009) (Table 6). The relationship between symptomatic ICH and the NLR, PLR, and CAR were evaluated and it was observed that an increase in the NLR and PLR values increased the risk of symptomatic ICH (*p* = 0.013, 0.009, respectively). No statistically significant relationship was observed between the CAR and symptomatic ICH (*p* = 0.784) (Table 7). While the factors affecting mortality were examined, it was observed that the inflammatory parameters NLR and PLR had an effect on mortality (*p* < 0.001, 0.003, respectively) (Table 8) but just in the univariate analyses. In the multivariate analyses, this result was not significant. The NLR, PLR, and CAR were not associated with ICH and mortality according to the multivariable logistic regression analysis. It is essential to mention this because these immune markers seem to be related to mortality and hemorrhage in previous literature, but, in our study, we demonstrated that there is no significant relationship at all.

**Figure 1:**
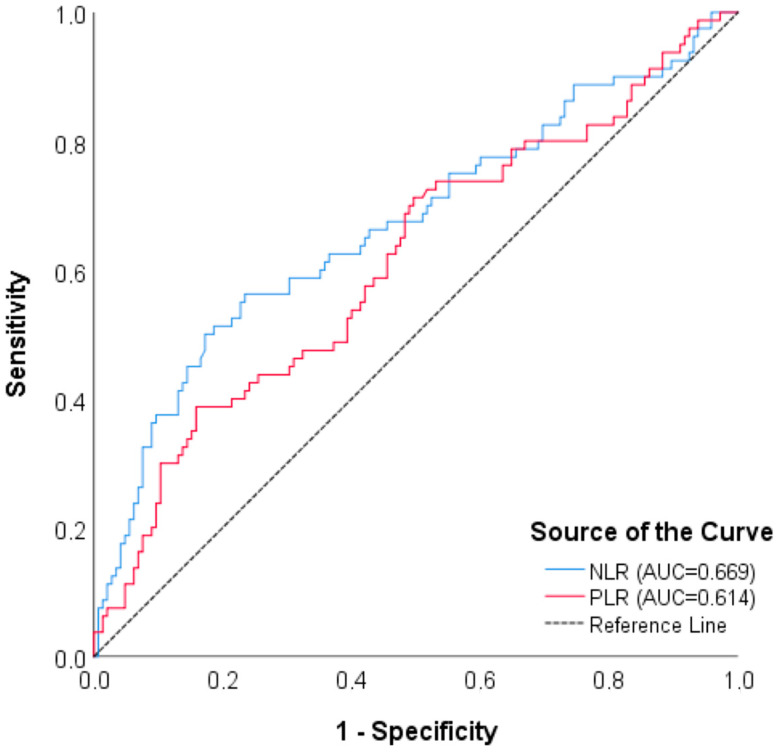
ROC curve for NLR and PLR—mortality.

**Figure 2:**
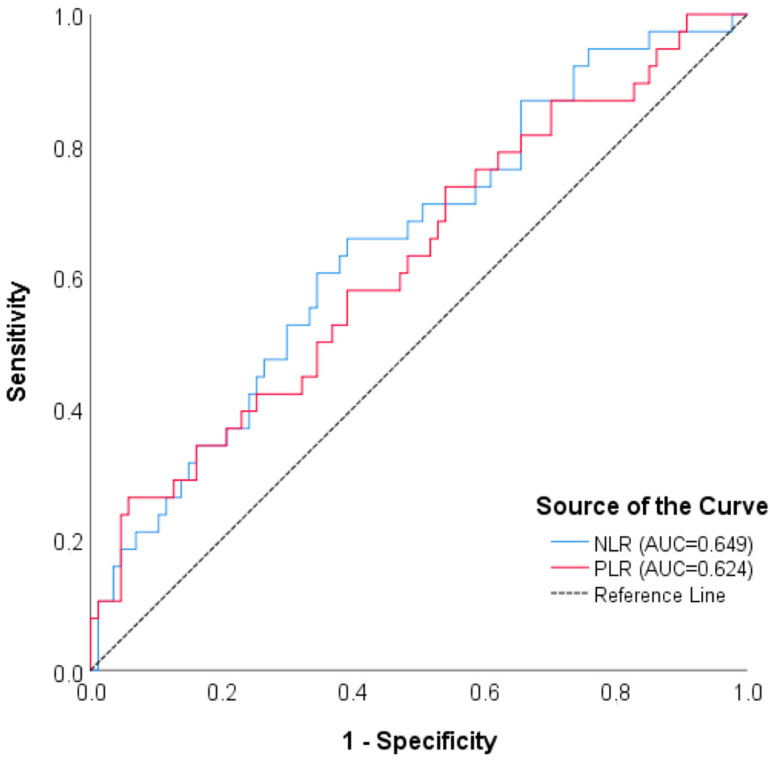
ROC curve for NLR and PLR—symptomatic ICH.

A cut-off value of the NLR parameter was found for ICH (*p* = 0.015) according to the ROC analyses of the inflammatory parameter evaluation to distinguish bleeding and mortality. For mortality, a cut-off value of the NLR and PLR values was obtained (<0.001, 0.005, respectively) (Table 9 and Table 10, Figure 1 and Figure 2). But the multivariate analyses did not support this effect. Compared to clinically proven scores such as mTICI, MRS, and NIHSS, the relationship between these immune markers and hemorrhage and mortality is weaker. In fact, unlike the univariate analyses, our findings indicate that these markers have no clinical significance according to multivariate analyses.

## 4. Discussion

EVT in acute ischemic stroke is proven to be efficient both for mortality and morbidity. However, sometimes, reperfusion is not adequate even when total recanalization is achieved. There are some causes of this situation related to both procedural and nonprocedural processes. The nonprocedural process of bad outcomes seems to be related to inflammation, as mentioned in the previous literature. While seeking appropriate markers, the investigators found some cheap and easily evaluated laboratory parameters such as the NLR, PLR, and CAR [10,11].

Inflammation plays an important role in the underlying mechanisms that cause early neurological deterioration, such as hemorrhagic transformation and brain edema, which develop after ischemic stroke [13,14]. After ischemic stroke, following the reactivation of macrophages, the disruption of the blood–brain barrier and migration of peripheral immune cells to the ischemic area begins. Cytotoxic mediators produced by leukocytes during the acute phase cause increased capillary permeability, the development of edema, hemorrhagic transformation after endothelial damage, and the exacerbation of ischemia. In addition, ischemic stroke induces lymphopenia through mechanisms involving complex neurohormonal responses, and this contributes to hemorrhagic transformation [13,14,15,16]. One of the most important results of our study is that, although the NLR values were observed to be associated with intracranial hemorrhage and mortality after EVT in acute stroke in univariate analysis, it was observed that it was not an indicator of hemorrhage and mortality when evaluated multivariably with many factors such as age and NIHSS and ASPECT scores. Therefore, unlike previous studies, it can be considered that the NLR is not a very strong prognosis predictor [15,17]. Many recent studies have examined the relationship of NLR, PLR, and CAR values with prognosis, mortality, and morbidity in cerebrovascular disease [15,17,18,19,20]. Pikija et al., in a study examining 187 patients who underwent EVT for acute ischemic stroke, found the NLR to be an independent predictor of the development of intracranial hemorrhage after EVT [16]. In a study by Lattanzi et al., high NLR values were found to be associated with early neurological deterioration [15].

Abnormalities in platelet function in acute ischemic stroke are seen with inflammation. Activated platelets interact with platelet-binding T lymphocyte cells, causing the production of cytokines, interferons, and chemokines, and prevent the healing of ischemia by changing adhesion molecules. In addition, reactive oxygen species that occur with ischemia increase the risk of hemorrhagic transformation. In many studies, an increased PLR has been associated with the risk of mortality in ischemic stroke both after IV-tPA and after EVT [16,18,20,21,22,23]. Although many studies conducted to date support the relationship between the PLR and symptomatic ICH, in a review by Sharma et al., the contradiction between the PLR and ICH was mentioned and it was argued that the etiology of ischemic stroke may affect the relationship between the PLR and ICH [18]. Diestro et al. reported that the PLR was not significantly associated with symptomatic ICH. Again, Eren et al. studied the relationship between the PLR and hemorrhagic transformation after IV-tPA in acute ischemic stroke and no connection was observed. Similar results were found in a study by İnanç et al. [18,24,25,26]. In our study, while no relationship was observed between the PLR and all types of intracranial hemorrhage, a relationship was observed between the PLR and symptomatic intracranial hemorrhage. Based on this, we thought that the PLR could be a marker of clinical worsening in patients with symptomatic ICH. ICH may also occur in ischemic stroke due to etiology or other causes even without IV-tPA and EVT. Even though these biomarkers seem to be related to mortality and hemorrhage according to our results, the AUC values have moderate predictive ability.

Another important result of our study is that there was no relationship between the CAR values and intracranial hemorrhage and mortality, unlike other studies in the literature. The CAR is the ratio of CRP to albumin, and rising CRP levels and decreasing albumin concentrations cause an increase in the CAR. Interleukin-6 secreted after stroke causes CRP production. Secreted CRP ensures the removal of damaged cells. Albumin is an important protein in the human body and its blood levels reflect the nutritional status. It is also a negative acute phase reactant. With inflammation, hepatic protein synthesis changes and albumin production decreases [10,27].

The relationship of the CAR with prognosis and mortality in many diseases, including stroke, has been studied [19,28,29]. It was noteworthy that, in our study, contrary to these studies, the CAR values were found to be unrelated to ICH and mortality. Similar results were found in a study conducted by Acır et al., which included 139 patients [30]. Previous studies have argued that the lack of a significant relationship in hemorrhagic stroke patients suggests that hemorrhagic strokes may involve different pathological processes, and that the prognostic importance of the CAR is less clear compared to its role in ischemic strokes [31]. Our study has an impact on this evaluation according to our findings.

In addition to these findings, our study has some limitations. First of all, this is a single-center study. Although the number of patients seems to be much higher than that in similar studies in this field, we recommend multicenter studies following the unique findings of our study. Secondly, only patients hospitalized in the Neurology Intensive Care Unit were included in this study. Patients hospitalized in other intensive care units or transferred elsewhere due to a lack of space were not included in the study. Another limitation is that the indications and procedure techniques of the EVT practitioners were not clearly documented. We also did not have the opportunity to evaluate the correlation between etiological factors and inflammation because of the number of patients referred to us from other districts or cities. Our study included 225 patients, but the number of symptomatic ICH cases (n = 38) and mortality events (n = 80) suggests potential power limitations for our subgroup analyses. The relatively low number of symptomatic hemorrhages may have affected the relationship between the CAR and prognosis. Also, analyzing whether the predictive value of the NLR, PLR, and CAR differed between the patients treated with EVT alone and the patients receiving IV-tPA in combination with EVT would have provided valuable information about potential interactions between systemic thrombolysis and these inflammatory markers. But the referred patients were an important aspect in our study. We do not know when they received IV-tPA, why they did not receive it if they did not receive it, or whether they completed this treatment, so we did not use these data as they may not have been reliable. The evaluation of other well-established scores such as the Systemic Inflammation Index (SII) and Systemic Inflammatory Response Index (SIRI) or biomarkers such as interleukin-6 (IL-6), interleukin-1 (IL-1), and D-dimer could have had an impact on our findings, but our study did not include these data.

## 5. Conclusions

Our findings in a medium-sized retrospective cohort highlight the need to better understand the post-stroke immune response and confirm that the NLR and PLR can be used as potential biomarkers in post-EVT mortality, the NLR in ICH, and the PLR in symptomatic ICH. In addition, the fact that similar findings to those of previous research regarding the CAR were not obtained in our study suggests that different mechanisms may be involved. It is important to understand the underlying mechanisms of inflammation in order to predict prognosis. Compared to clinically proven scores such as mTICI, mRS, and NIHSS, the relationship between these immune markers and hemorrhage and mortality is weaker. In fact, unlike the univariate analyses, our findings indicate that these markers have no clinical significance according to multivariate analyses.

All of these biomarkers seem to be related to inflammation in stroke-related hemorrhage and mortality, but because of the nature of inflammation, which can be affected by various factors, these biomarkers are not as valuable as mTICI, mRS, and NIHSS scores.

## Figures and Tables

**Table 1:** Demographic findings.

	Average ± ss	Median (IQR)
Age	66.95 ± 12.74	68 (58–76)
NIHSS	14.1 ± 4.94	15 (10–18)
mRS	3.8 ± 1.95	4 (2–6)
	n	%
Gender		
Male	133	59.1
Female	92	40.9
ASPECTS		
0–6	66	29.3
7–10	159	70.7
TOAST classification		
Large vascular disease	67	29.8
Cardioembolic	115	51.1
Due to other reasons	4	1.8
Undetermined cause	39	17.3
mTICI		
0	28	12.4
1	15	6.7
2a	34	15.1
2b	45	20
2c	16	7.1
3	87	38.7
Presence of ICH		
No	100	44.4
Yes	125	55.6
ICH type		
Hemorrhagic transformation Type 1	56	24.9
Hemorrhagic transformation Type 2	39	17.3
Parenchymal hematoma type 1	15	6.7
Parenchymal hematoma type 2	15	6.7
Symptomatic ICH		
No	87	69.6
Yes	38	30.4
IV-tPA		
No	47	32.9
Yes	96	67.1
mRS		
Good outcome	61	27.1
Poor outcome	164	72.9
Mortality		
Death	80	35.6
Survivors	145	64.4
Recanalization area		
Basilary artery	22	9.8
Right MCA	66	29.3
Left MCA	70	31.1
Right ICA	28	12.4
Left ICA	39	17.3

IQR: interquartile range, NIHSS: National Institutes of Health Stroke Scale, mRS: modified Rankin Score, TOAST: Trial of ORG 10172 in Acute Stroke Treatment, ASPECTS: Alberta Stroke Program Early CT Score, mTICI: Treatment In Cerebral Infarction, ICH: intracranial hemorrhage, IV-tPA: intravenous thrombolysis, MCA: middle cerebral artery, ICA: internal carotid artery.

**Table 2:** The connection between ICH and categorical variables.

	ICH (−)	ICH (+)	Total	Test Statistics	*p* Value
Gender					
Male	50 (50)	83 (66.4)	133 (59.1)	6.182	0.013^x^
Female	50 (50)	42 (33.6)	92 (40.9)		
ASPECTS					
0–6	25 (37.9)	41 (62.1)	66 (29.3)	1.631	0.202 ^x^
7–10	75 (47.2)	84 (52.8)	159 (70.7)		
Recanalization area					
Basilar artery	13 (13)	9 (7.2)	22 (9.8)	3.807	0.433 ^x^
Right MCA	26 (26)	40 (32)	66 (29.3)		
Left MCA	29 (29)	41 (32.8)	70 (31.1)		
Right ICA	15 (15)	13 (10.4)	28 (12.4)		
Left ICA	17 (17)	22 (17.6)	39 (17.3)		
mRS					
Good outcome	36 (36)	25 (20)	61 (27.1)	7.197	0.007 ^x^
Poor outcome	64 (64)	100 (80)	164 (72.9)		
Mortality					
Death	21 (21)	59 (47.2)	80 (35.6)	16.643	<0.001 ^x^
Survivors	79 (79)	66 (52.8)	145 (64.4)		

^x^ Pearson’s Chi-square test; n (%). ICH: intracranial hemorrhage, ASPECTS: Alberta Stroke Program Early CT Score, MCA: middle cerebral artery, ICA: internal carotid artery, mRS: modified Rankin Score.

**Table 3:** Comparison of quantitative data according to ICH groups.

	ICH (−)	ICH (+)	Test Statistics	*p*
Age (years)	68 (30–92)	68 (41–89)	6077.000	0.722 ^x^
NIHSS	14 (4–24)	15 (3–24)	5422.500	0.088 ^x^
Neutrophil	8.29 (3.14–21.34)	9.7 (1.9–18.34)	4907.000	0.006 ^x^
Lymphocyte	1.22 (0.2–5.58)	1.1 (0.2–9.8)	6981.500	0.132 ^x^
Platelet	211 (113–647)	229 (83–543)	5826.000	0.383 ^x^
CRP	7.9 (0.3–269.5)	8.7 (0.9–221.1)	6264.500	0.977 ^x^
Albumin	35.69 ± 4.52	36.59 ± 4.3	−1.518	0.131 ^y^
NLR	6.75 (0.97–41.41)	9.29 (0.96–49.67)	5068.500	0.015 ^x^
PLR	177.73 (31.18–779.59)	188.82 (17.35–1175)	5463.500	0.105 ^x^
CAR	0.22 (0.01–7.25)	0.24 (0.02–6.5)	6364.000	0.815 ^x^

^x^ Mann–Whitney U test; ^y^ independent two-sample *t* test; mean ± s. deflection; median (minimum–maximum). ICH: intracranial hemorrhage, NIHSS: National Institutes of Health Stroke Scale, CRP: C-reactive protein, NLR: neutrophil/lymphocyte ratio, PLR: platelet/lymphocyte ratio, CAR: CRP/albumin ratio.

**Table 4:** Comparison of NLR, PLR, and CAR values according to mortality.

	Death	Survivors	Test Statistics	*p*
Age (years)	69.3 ± 12	65.65 ± 12.99	−2.073	0.039 ^x^
NIHSS	18 (5–24)	13 (3–24)	2833.500	<0.001 ^y^
Neutrophil	10.4 (1.9–18)	8.5 (3.14–21.34)	4196.500	<0.001 ^y^
Lymphocyte	0.99 (0.2–3.9)	1.25 (0.2–9.8)	7232.500	0.002 ^y^
Platelet	224.5 (83–647)	219 (114–507)	5682.000	0.802 ^y^
CRP	36.9 (20.9–44.4)	36.5 (17.6–48.1)	5844.000	0.926 ^y^
Albumin	35.69 ± 4.52	36.59 ± 4.3	−1.518	0.131 ^y^
NLR	11.64 (2.04–47.67)	6.99 (0.96–49.67)	7760.500	<0.001 ^x^
PLR	207.28 (69.49–1175)	171.43 (17.35–779.59)	7119.500	0.005 ^x^
CAR	0.27 (0.02–3.66)	0.21 (0.01–7.25)	6620.500	0.079 ^x^

^x^ Mann–Whitney U test; ^y^ independent two-sample *t* test; mean ± s. deflection; median (minimum-maximum). NIHSS: National Institutes of Health Stroke Scale, CRP: C-reactive protein, NLR: neutrophil/lymphocyte ratio, PLR: platelet/lymphocyte ratio, CAR: CRP/albumin ratio.

**Table 5:** Comparison of NLR, PLR, and CAR values according to mRS groups.

	Good Outcome	Poor Outcome	Test Statistics	*p*
NLR	5.58 (0.96–17.83)	9.2 (0.97–49.67)	3238.500	<0.001 ^y^
PLR	132.86 (17.35–762.96)	205.9 (31.18–1175)	3164.500	<0.001 ^y^
CAR	0.19 (0.01–6.84)	0.24 (0.02–7.25)	4356.500	0.137 ^y^

^y^ Mann–Whitney U test; mean ± s. deflection; median (minimum–maximum). NLR: neutrophil/lymphocyte ratio, PLR: platelet/lymphocyte ratio, CAR: CRP/albumin ratio.

**Table 6:** Examination of risk factors affecting the development of ICH using binary logistic regression analysis.

	Univariate		Multiple	
	OR (%95 CI)	*p*	OR (%95 CI)	*p*
Neutrophil	1.112 (1.025–1.207)	0.011	1.054 (0.949–1.172)	0.325
Lymphocyte	0.866 (0.656–1.144)	0.310	1.079 (0.757–1.538)	0.673
NLR	1.056 (1.014–1.099)	0.009	1.059 (0.968–1.158)	0.212
PLR	1.002 (1–1.003)	0.081	1.001 (0.998–1.005)	0.459
CAR	0.859 (0.667–1.107)	0.241	0.834 (0.62–1.123)	0.232
ASPECTS	0.896 (0.766–1.048)	0.169	0.977 (0.811–1.176)	0.804
mTICI				
0	Reference			
1	1.5 (0.421–5.347)	0.532	1.626 (0.425–6.223)	0.478
2a	0.889 (0.327–2.419)	0.818	1.399 (0.471–4.154)	0.545
2b	1.5 (0.58–3.883)	0.403	3.082 (1.041–9.124)	0.042
2c	1.667 (0.475–5.842)	0.425	4.12 (0.992–17.109)	0.051
3	1.289 (0.549–3.027)	0.559	3.139 (1.134–8.687)	0.028
mRS	1.288 (1.118–1.483)	<0.001	1.374 (1.133–1.666)	0.001
NIHSS	1.047 (0.992–1.105)	0.094	1.01 (0.949–1.075)	0.751
Age	1.008 (0.988–1.029)	0.431	1.014 (0.99–1.039)	0.252

ICH: intracranial hemorrhage, NLR: neutrophil/lymphocyte ratio, PLR: platelet/lymphocyte ratio, CAR: CRP/albumin ratio, ASPECTS: Alberta Stroke Program Early CT score, mTICI: Treatment In Cerebral Infarction, mRS: modified Rankin Score, NIHSS: National Institutes of Health Stroke Scale.

**Table 7:** Examining the effect of NLR, PLR, and CAR on symptomatic ICH.

	Univariate		Multiple	
	OR (%95 CI)	*p*	OR (%95 CI)	*p*
NLR	1.058 (1.012–1.106)	0.013	1.013(0.931–1.103)	0.759
PLR	1.003 (1.001–1.005)	0.009	1.002 (0.998–1.006)	0.254
CAR	0.934 (0.573–1.522)	0.784	0.912 (0.543–1.53)	0.726

ICH: intracranial hemorrhage, NLR: neutrophil/lymphocyte ratio, PLR: platelet/lymphocyte ratio, CAR: CRP/albumin ratio.

**Table 8:** Examination of risk factors affecting mortality using binary logistic regression analysis.

	Univariate		Multiple	
	OR (%95 CI)	*p*	OR (%95 CI)	*p*
Neutrophil	1.148 (1.056–1.247)	0.001	0.942 (0.846–1.049)	0.275
Lymphocyte	0.618 (0.418–0.915)	0.016	1.034 (0.675 –1.584)	0.877
NLR	1.089 (1.045–1.136)	<0.001	0.924 (0.842–1.013)	0.092
PLR	1.003 (1.001–1.004)	0.003	1.003 (0.998–1.008)	0.182
CAR	1.072 (0.836–1.373)	0.584	0.857 (0.575–1.276)	0.446
ASPECTS	0.851 (0.725–1)	0.050	1.128 (0.84–1.515)	0.423
mTICI				
0	Reference			
1	0.172 (0.043–0.693)	0.013	0.075 (0.012–0.457)	0.005
2a	0.227 (0.078–0.661)	0.007	0.226 (0.046–1.118)	0.068
2b	0.153 (0.054–0.436)	<0.001	0.191 (0.042–0.863)	0.031
2c	0.158 (0.04–0.629)	0.009	0.801 (0.077–8.319)	0.853
3	0.262 (0.106–0.649)	0.004	1.503 (0.362–6.24)	0.575
mRS	3.254 (2.376–4.457)	<0.001	4.066 (2.599–6.361)	<0.001
NIHSS	1.238 (1.151–1.332)	<0.001	1.289 (1.147–1.448)	<0.001
Age	1.024 (1.001–1.047)	0.041	1.03 (0.991–1.069)	0.130

NLR: neutrophil/lymphocyte ratio, PLR: platelet/lymphocyte ratio, CAR: CRP/albumin ratio, ASPECTS: Alberta Stroke Program Early CT score, mTICI: Treatment in Cerebral Infarction, mRS: modified Rankin Score, NIHSS: National Institutes of Health Stroke Scale.

**Table 9:** ROC analysis results of parameters in distinguishing ICH and mortality.

		AUC (%95 CI)	*p*	Cut-Off	Sensitivity	Specificity	PPV (%)	NPV (%)
ICH	NLR	0.595 (0.521–0.668)	0.015	10.12	45.6%	73%	67.8%	51.7%
	PLR	0.563 (0.488–0.638)	0.105	---	---	---	---	---
	CAR	0.491 (0.415–0.567)	0.816	---	---	---	---	---
Mortality	NLR	0.669 (0.592–0.746)	<0.001	10.53	56.2%	76.5%	56.9%	76%
	PLR	0.614 (0.535–0.692)	0.005	278.41	38.7%	84.1%	57.4%	71.3%
	CAR	0.571 (0.491–0.651)	0.078	---	---	---	---	---

PPV: positive predictive value, NPV: negative predictive value, direction of the test (≥), ICH: intracranial hemorrhage, NLR: neutrophil/lymphocyte ratio, PLR: platelet/lymphocyte ratio, CAR: CRP/albumin ratio. ---: For non-significant results, the “cut off” value could not be calculated and is therefore marked with “---”.

**Table 10:** ROC analysis results of NLR and PLR values in distinguishing symptomatic ICH.

	AUC (%95 CI)	*p*	Cut-Off	Sensitivity	Specificity	PPV (%)	NPV (%)
NLR	0.649 (0.545–0.752)	0.008	9.833	65.79%	60.92%	42.37%	80.30%
PLR	0.624 (0.516–0.732)	0.028	445.714	26.32%	94.25%	66.67%	74.55%

PPV: positive predictive value, NPV: negative predictive value, direction of the test (≥), ICH: intracranial hemorrhage, NLR: neutrophil/lymphocyte ratio, PLR: platelet/lymphocyte ratio.

## Data Availability

The original contributions presented in the study are included in this article; further inquiries can be directed to the corresponding author.

## Abbreviations

The following abbreviations are used in this manuscript:

LVO	Large vessel occlusion
EVT	Endovascular therapies
NLR	Neutrophil/lymphocyte ratio
PLR	Platelet/lymphocyte ratio
CAR	CRP/albumin ratio
ICA	Internal carotid artery
MCA	Middle carotid artery
CT	Computerized tomography
NIHSS	National Institutes of Health Stroke Scale
IV-tPA	Intravenous thrombolytic therapy
ASPECTS	Alberta Stroke Program Early CT Score
mRS	Modified Rankin Score
ICH	Intracranial hemorrhage
TOAST	Trial of ORG 10172 in Acute Stroke Treatment
mTICI	Treatment In Cerebral Infarction
